# Probability density function for random photon steps in a binary (isotropic-Poisson) statistical mixture

**DOI:** 10.1038/s41598-023-36919-2

**Published:** 2023-06-19

**Authors:** Tiziano Binzoni, Alain Mazzolo

**Affiliations:** 1 Department of Radiology and Medical Informatics, University Hospital, Geneva, 1211 Switzerland; 2 Université Paris-Saclay, CEA, Service d’Études des Réacteurs et de Mathématiques Appliquées, 91191 Gif-sur-Yvette, France

**Keywords:** Mathematics and computing, Optics and photonics

## Abstract

Monte Carlo (MC) simulations allowing to describe photons propagation in statistical mixtures represent an interest that goes way beyond the domain of optics, and can cover, e.g., nuclear reactor physics, image analysis or life science just to name a few. MC simulations are considered a “gold standard” because they give exact solutions (in the statistical sense), however, in the case of statistical mixtures their implementation is often extremely complex. For this reason, the aim of the present contribution is to propose a new approach that should allow us in the future to simplify the MC approach. This is done through an explanatory example, i.e.; by deriving the ‘exact’ analytical expression for the probability density function of photons’ random steps (single step function, SSF) propagating in a medium represented as a binary (isotropic-Poisson) statistical mixture. The use of the SSF reduces the problem to an ‘equivalent’ homogeneous medium behaving exactly as the original binary statistical mixture. This will reduce hundreds MC simulations, allowing to obtain one set of wanted parameters, to only one equivalent simple MC simulation. To the best of our knowledge the analytically ‘exact’ SSF for a binary (isotropic-Poisson) statistical mixture has never been derived before.

## Introduction

Two probability density functions (pdf) are at the heart of Monte Carlo simulations describing photon propagation in biomedical optics, optics or, in general, particle transport in diffusive media: the “phase function” (PF) and the pdf allowing to describe the probability density for a photon to reach a distance *s* in the medium, without interactions. For simplicity, we will call the latter pdf “single step function” (SSF). The knowledge of the SSF is fundamental because it is mandatory for the implementation of a MC simulation, and because from the SSF we can extract the main characteristics of photon propagation through the medium^1^.

The specific shape of the SSF is determined, in a very complex way, by the physical characteristics of the investigated media. For this reason, in the majority of the cases, the SSF cannot be derived theoretically, but is estimated from experimental data. Among the different SSFs appearing in the literature, one in particular seems to be more recurrent and applicable in many situations; i.e., the one derived from the so called Beer–Lambert–Bouguer law, with pdf

1 $$p_{{LB}} (s;\mu _{t} ) = \mu _{t} e^{{ - \mu _{t} s}} ,$$

where $\mu _t$ is the extinction coefficient of the medium. Historically, the SSF $p_{LB}(.)$ has been determined experimentally and, nowadays, its applicability covers a panoply of different domains^2–4^.

**Figure 1:**
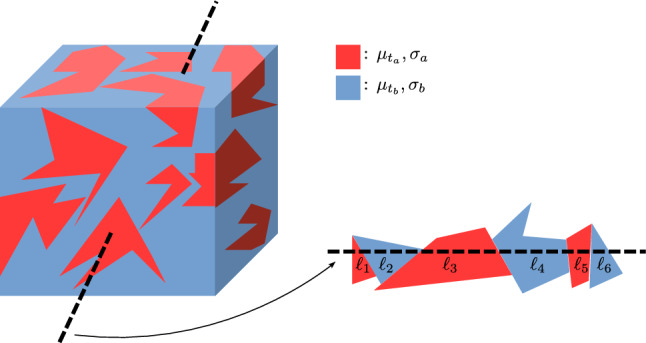
Schematic of a 3D medium (the cubic shape is just for simplicity, and may also be infinite) composed by random tessels of two kinds of materials (red and sky blue) satisfying both Eq. (1), with $\mu _{t_a}$ and $\mu _{t_b}$. The random oriented dashed line crosses different areas of random lengths $\ell _1, \ell _1,\ldots ,\ell _6$ (along the line), satisfying Eq. (2), with parameters $\sigma _a$ and $\sigma _b$. If Eq. (2) with parameters $\sigma _a$ and $\sigma _b$ always holds for any dashed random oriented line, the medium is called isotropic.

Due to the large number of physical systems that can be described by $p_{LB}(.)$, some caution may be natural when describing systems with an SSF that appears to deviate from the Eq. (1). In fact, the question may arise whether SSFs different from Eq. (1) are not simply the result generated by compound media of immiscible materials; where each bunch of material — considering the subject to come in the manuscript, we will call them “tessels”—always satisfies the classical Beer–Lambert–Bouguer law (Eq. 1), with their own $\mu _t$ (for an intuitive example see the schematic in Fig. 1).

With this question in mind, many studies have already been proposed in the literature; going from numerical MC simulations to theoretical approaches^5–8^. Interestingly, this approach also applies to image rendering^9^.

Due to their ability to describe actual physical systems, a particular attention has been given to “random” media obeying mixing statistics. In intuitive words, random media similar to the one appearing in Fig. 1 were generated with the desired statistical law. Then, the exact or approximated physical quantities of interest (e.g., in the present context the SSF) were obtained (theoretically or numerically) on many random realizations of the medium and, finally, the “average value” of each quantity was derived. Unfortunately, the common characteristic of these studies is that they never propose an *exact* analytical solution for the SSF. This is why, the aim of the present contribution is to derive an exact analytical solution for a well celebrated model: the binary (isotropic-Poisson) statistical mixture.

But, why the knowledge of an exact analytical expression for the SSF may represent any advantage? One of the reasons is that repeated MC simulations of random media, sometimes composed by thousands of tessels of complex shapes, may be extremely computational expensive; in particular if simulations must be repeated a large number of times. The knowledge of an analytical expression for SSF, describing our random medium with mixing statistics, allows one, in principle, to perform *only one simulation* on a equivalent *homogeneous medium*, and to obtain the same desired quantities (e.g., transmitted or reflected photon fluxes) as for the original problem.

## Theory

### Isotropic Poisson tesselation with a binary mixture

**Figure 2:**
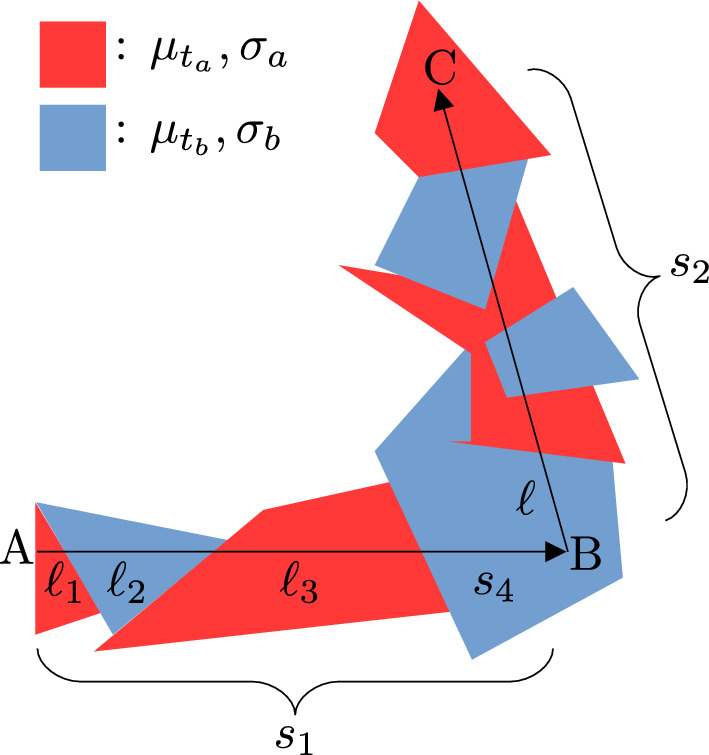
Simplified 2D schematic representing a typical case of photon propagation in a (binary) medium. A, entrance point in the medium; B, the photon is scattered and changes its direction; C, the photon is absorbed and stops its propagation. $s_1$, first step length; $s_2$, second step length. In the present theoretical context and in MC simulations $s_1$ and $s_2$ are decomposed in sub-steps; e.g., $s_1=\ell _1+\ell _2+\ell _3+s_4$. Note that $s_4$ is shorter than the tessel length because the photon is scattered in B. The parameter $\ell$ is the distance from point B to the next (red) tessel.

The three dimensional random medium that we will consider in the present contribution, is a medium where a photon that propagates in a straight line, crosses, alternatively, two kind of homogeneous material (tessels) (see, e.g., Fig. 2). The length of each piece of path that crosses a tessel (e.g., $\ell _1, \ell _2, \ell _3$ in Fig. 2)  is given by a probabilistic law. If the probabilistic law remains the same for any direction of propagation of the photon, then the medium is called isotropic. It has been demonstrated that if we want an isotropic medium with the above characteristics, then the probabilistic law must be exponential; i.e.^6,10^,

2 $$\begin{aligned} p_{Tes}(\ell ;\sigma )=\sigma e^{-\sigma \ell }, \end{aligned}$$

where $\ell$ is the (random) length of the tessel in the direction of the photon propagation, and the constant $\sigma \in \{\sigma _a,\sigma _b\}$, depending on the type of tessel we want to generate (red or sky blue in Fig. 2). Observe that the mean tessel length is

3 $$\begin{aligned} \langle \ell \rangle =\int _0^{+\infty } \ell p_{Tes}(\ell ;\sigma ) d\ell =\frac{1}{\sigma }. \end{aligned}$$

Moreover, to have an isotropic medium, $\sigma _a$ and $\sigma _b$ must satisfy the following relationship^6,10^

4 $$\begin{aligned} \sigma _a=\frac{1}{L}(1- P ), \end{aligned}$$

and

5 $$\begin{aligned} \sigma _b=\frac{1}{L} P, \end{aligned}$$

where $(1-P)$ is the probability to have a tessel of type “*a*”, and *P* the probability to have a tessel of type “*b*”. The constant *L* is a parameter utilized in the construction of the medium. For such a medium in the literature we speak about “isotropic Poisson tesselation with a binary mixture”.

### Single step function: exact analytical derivation

In this section we will analytically derive the exact SSF, $p_{s_{\textrm{mix}}}(s;\mu _{t_a},\mu _{t_b},\sigma _a,\sigma _b)$, for the isotropic Poisson tesselation with a binary mixture model; where $\mu _{t_a}$ and $\mu _{t_b}$ are the extinction coefficients of the tessels of type “a” and “b”, respectively. The random step *s* (ballistic propagation) is defined as a straight line of random length, going from the starting point to the point where the photon is absorbed or scattered. Thus, a step may go across many tessels (e.g., steps $s_1$ or $s_2$ in Fig. 2). By following the approach presented e.g. in Refs.^1,11^ a step can be decomposed as a sum of sub-steps lengths—independently generated—covered by the photon in the “a” and “b” regions. To obtain $p_{s_{\textrm{mix}}}(.)$ (Eq. 32) we need before to derive some intermediate functions.

#### Main points of the procedure

1. From the Beer–Lambert–Bouguer law $p_{LB}(.)$ (Eq. 1) and the probabilistic law describing the tessel length distribution $p_{Tes}(.)$ (Eq. 2) we derive the probability for a photon to make a step larger than *s*, $P_{>s}(.)$ (Eq. 6), and the probability for a photon to make a step larger than a random tessel of length $s'$, $P_1(.)$ (Eq. 7).
2. $P_1(.)$ allows one to derive the probability for a photon to make *N* consecutive steps larger than the corresponding *N* consecutive random tessel lengths, $P_{\textrm{N}}(.)$ (Eq. 10).
3. From $p_{LB}(.)$ (Eq. 1) and $p_{Tes}(.)$ (Eq. 2) we derive the pdf $p_{s_0}(.)$ (Eq. 12) for a photon step of random lengh *s* to remain inside a tessel of random length $\ell$.
4. The probability $p_{Tes_1}(.)ds$ (Eq. 14) for a photon to make $N=1$ steps is obtained by using again $p_{LB}(.)$ (Eq. 1) and $p_{Tes}(.)$ (Eq. 2).
5. $p_{Tes_1}(.)$ is the key pdf allowing us to derive the probability $p_{Tes_N}(.)ds$ (Eqs. 22 and 29) for a photon to make a step of length *s*, in exactly *N* substeps, equal to the sum of the lengths of the *N* tessels.
6. The pdf $p_{s_N}(.)$ (Eq. 30) for a photon to jump over *N* tessels and reach a distance *s* is derived from $p_{s_0}(.)$ and $p_{Tes_N}(.)$.
7. The pdf $p_s(.)$ (Eq. 31) that allows one to describe the photon steps *s* in the subset of the medium realizations where the photon always starts from a tessel of type “a” (e.g. red tessel in Fig. 2) and, independently, in the subset of type “b”, can be expressed using $p_{s_N}(.)$ and $P_{\textrm{N}}(.)$.
8. Finally, the pdf $p_{s_{\textrm{mix}}}(.)$ (Eq. 32), representing the main result can be expressed as the weighted sum of two terms in $p_s(.)$. The weight parameters are the probability to have a tessel of type “a” ($1-P$) and the probability to have a tessel of type “b” (*P*).

**Figure 3:**
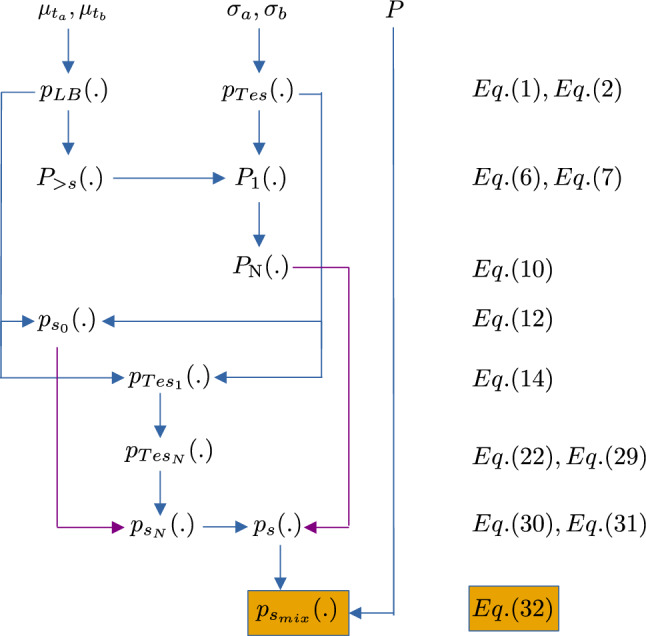
Diagram summarizing the main steps allowing to derive the SSF $p_{s_{\textrm{mix}}}(s;\mu _{t_a},\mu _{t_b},\sigma _a,\sigma _b)$.

This is what is done in the following sub-sections. The above points are schematically summarized in Fig. 3.

Complex analytical calculations were performed using Mathematica® software.

#### Probability mass function $P_{\textrm{N}}(.)$

The probability for a photon to make a step larger than *s* is

6 $$\begin{aligned} P_{>s}(s;\mu _t)= 1-\int _0^{s}p_{LB}(s';\mu _t) ds'=e^{-\mu _t s}. \end{aligned}$$

The probability for a photon to make a step larger than a random tessel of length $s'$ is (the photon starts at the tessel boundary)

7 $$\begin{aligned} P_1(\mu _t,\sigma )&=\int _0^{+\infty }P_{>s}(s';\mu _t)p_{Tes}(s';\sigma )ds' =\frac{\sigma }{\sigma +\mu _t}. \end{aligned}$$

Considered the fact that the tessels crossed by a photon (ballistic propagation) have alternate $\mu _{t_a}$ and $\mu _{t_b}$ values, the probability to make a series of consecutive steps larger than the corresponding random tessel lengths must be of the form

8 $$\begin{aligned} P_1(\mu _{t_a},\sigma _a)P_1(\mu _{t_b},\sigma _b)P_1(\mu _{t_a},\sigma _a)P_1(\mu _{t_b},\sigma _b)P_1(\mu _{t_a},\sigma _a)\ldots \end{aligned}$$

Therefore, the probability $P_{\textrm{N}}(\mu _{t_a},\mu _{t_b},\sigma _a,\sigma _b,N)$ for a photon to make *N* *consecutive* steps larger than the corresponding *N* *consecutive* random tessel lengths (the first tessel always has $\mu _{t_a}$) is

9 $$\begin{aligned}{}&P_{\textrm{N}}(\mu _{t_a},\mu _{t_b},\sigma _a,\sigma _b,N) \nonumber \\&\quad =\left\{ \begin{array}{lll} 1-P_1(\mu _{t_a},\sigma _a) &{} \text{ if } N=0 \\ \\ \left\{ \left[ P_1(\mu _{t_a},\sigma _a) P_1(\mu _{t_b},\sigma _b)\right] ^\frac{N}{2} \right\} \left[ 1-P_1(\mu _{t_a},\sigma _a)\right] &{} \text{ if } N \text{ even } \\ \\ \left\{ P_1(\mu _{t_a},\sigma _a) \left[ P_1(\mu _{t_a},\sigma _a)P_1(\mu _{t_b},\sigma _b)\right] ^\frac{(N-1)}{2}\right\} \left[ 1-P_1(\mu _{t_b},\sigma _b)\right] &{} \text{ if } N \text{ odd }, \end{array} \right. \end{aligned}$$

where $\sum _{N=0}^{+\infty }{P_{\textrm{N}}}(\mu _{t_a},\mu _{t_b},\sigma _a,\sigma _b,N)=1$, as expected.

In other words, if $N=0$ (first line Eq. (9)), the photon makes a step shorter than the random length of the first tessel. If *N* is even, then the last term in Eq. (8) is $P_1(\mu _{t_b},\sigma _b)$. If *N* is odd, the last term in Eq. (8) is $P_1(\mu _{t_a},\sigma _a)$. This explains the terms in curly brackets in the second and third line of Eq. (9). The last photon step always has probability $1-P_1(\mu _{t_a},\sigma _a)$ or $1-P_1(\mu _{t_b},\sigma _b)$, $\forall N$ (Eq. 9), because it is where the photon stops (i.e., the last step must be shorter than the last random tessel length). By inserting Eq. (7) in Eq. (9) we find

10 $$\begin{aligned}{}&{P_{\textrm{N}}}(\mu _{t_a},\mu _{t_b},\sigma _a,\sigma _b,N) \nonumber \\&\quad =\left\{ \begin{array}{lll} \frac{\mu _{t_a}}{\mu _{t_a+\sigma _a}} &{} \text{ if } N=0 \\ \\ \frac{\mu _{t_a} (\sigma _a\sigma _b)^\frac{N}{2}}{(\mu _{t_a}+\sigma _a)^{\frac{N+2}{2}}(\mu _{t_b}+\sigma _b)^{\frac{N}{2}}} &{} \text{ if } N \text{ even } \\ \\ \frac{\mu _{t_b} \sigma _a^\frac{N+1}{2} \sigma _b^\frac{N-1}{2}}{(\mu _{t_a}+\sigma _a)^{\frac{N+1}{2}}(\mu _{t_b}+\sigma _b)^{\frac{N+1}{2}}} &{} \text{ if } N \text{ odd }. \end{array} \right. \end{aligned}$$

We derive here the pdf $p_{s_0}(s;\mu _t,\sigma )$ for a photon step of random lengh *s* to remain inside a tessel of random length $\ell$ (the photon always starts on the tessel boundary). To do this, we apply an upper cut-off to Eq. (1) at the distance $\ell$, i.e.,

11 $$\begin{aligned} p_{LB_{s<\ell }}(s;\mu _t,\ell )= \Theta (\ell -s) \mu _t e^{-\mu _t s}, \end{aligned}$$

where $\Theta (.)$ is the Heaviside function. Thus, the pdf $p_{s_0}(s;\mu _t,\sigma )$ is obtained as

12 $$\begin{aligned} p_{s_0}(s;\mu _t,\sigma )= \frac{ \int _0^{+\infty }p_{LB_{s<\ell }}(s;\mu _t,\ell )p_{Tes}(\ell ;\sigma )d\ell }{ \int _0^{+\infty }\int _0^{+\infty }p_{LB_{s<\ell }}(s;\mu _t,\ell )p_{Tes}(\ell ;\sigma )d\ell ds}= (\mu _t+\sigma )e^{-(\mu _t+\sigma )s}, \end{aligned}$$

where the denominator appearing of Eq. (12) is the normalization factor.

We need also the probability $p_{Tes_N}(s;\mu _{t_a},\mu _{t_b},\sigma _a,\sigma _b,N)ds$ for a photon to make $N>1$ steps, of total random length $\ell _1+\ell _2+\cdots +\ell _N=s\in \left[ s-\frac{ds}{2},s+\frac{ds}{2}\right]$; where $\ell _i$ is the (random) length of the $i\textrm{th}$ tessel, in the photon direction (the function $p_{Tes_N}(.)$ is a pdf). To this aim, we start with the pdf $p_{Tes_1}(s;\mu _t,\sigma _t)$ for a single tessel (case $N=1$).

#### Probability density function $p_{Tes_N}(.)$: case $N=1$

By applying a lower cut-off at the distance $\ell -\frac{\Delta \ell }{2}$ and an upper cut-off at the distance $\ell +\frac{\Delta \ell }{2}$ to Eq. (1) ($0<\Delta \ell \ll 1$), we express the fact that we want the photon falls at a distance $\ell$ (tessel length in the direction of the photon propagation), i.e.,

13 $$\begin{aligned} p_{LB_{s\approx \ell }}(s;\mu _t,\ell )=\left\{ \Theta \left[ s-\left( \ell -\frac{\Delta \ell }{2}\right) \right] -\Theta \left[ s-\left( \ell +\frac{\Delta \ell }{2}\right) \right] \right\} \mu _t e^{-\mu _t s}. \end{aligned}$$

Thus

14 $$\begin{aligned} p_{Tes_1}(s;\mu _t,\sigma )\underset{N=1}{=}\ \lim _{\Delta \ell \rightarrow 0} \frac{ \int _0^{+\infty }p_{LB_{s\approx \ell }}(s;\mu _t,\ell )p_{Tes}(\ell ;\sigma )d\ell }{ \int _0^{+\infty }\int _0^{+\infty }p_{LB_{s\approx \ell }}(s;\mu _t,\ell )p_{Tes}(\ell ;\sigma )d\ell ds}= (\mu _t+\sigma )e^{-(\mu _t+\sigma )s}, \end{aligned}$$

where $\mu _t\in \{\mu _{t_a},\mu _{t_b}\}$ and where the denominator is the normalization factor allowing to obtain the pdf. Note that

15 $$\begin{aligned} p_{Tes_1}(s;\mu _t,\sigma )=p_{s_0}(s;\mu _t,\sigma ). \end{aligned}$$

Equation (14) allows one to treat the particular case of the pdf $p_{Tes_N}(s;\mu _t,\mu _t,\sigma ,\sigma ,N)$ of *N* consecutive tessels with same $\mu _t$ and $\sigma$; i.e.,

16 $$\begin{aligned}{}&p_{Tes_N}(s;\mu _t,\mu _t,\sigma ,\sigma ,N) \nonumber \\&\quad =\int _0^{+\infty }\ldots \int _0^{+\infty }\int _0^{+\infty } p_{Tes_1}(\ell _1;\mu _t,\sigma ) p_{Tes_1}(\ell _2;\mu _t,\sigma ) \ldots p_{Tes_1}(\ell _N;\mu _t,\sigma ) \nonumber \\&\quad \times \delta (\ell _1+\ell _2+\cdots +\ell _N-s) d\ell _1d\ell _2\ldots d\ell _N \nonumber \\&\quad = \frac{s^{N-1} (\mu _t+\sigma )^N e^{-(\mu _t+\sigma )s}}{(N-1)!}. \end{aligned}$$

This is the expected result since the pdf $p_{Tes}(.)$ is the sum of N positive random variables described by the same exponential pdf $p_{Tes_1}(.)$; resulting in a gamma function with parameters *N* and $\mu _t+\sigma$ (Eq. 16). Equation (16) allows one to consider two cases for $p_{Tes_N}(s;\mu _{t_a},\mu _{t_b},\sigma _a,\sigma _b,N)$; i.e., for *N* odd and *N* even.

Note that, in the present contribution the sum of the random variables, with the resulting relative pdf, are presented using a formalism implying the delta Dirac generalized function (see, e.g., Eqs. 16). However, the formalism involving the use of the characteristic functions (Laplace transform of the pdfs), to obtain the same results, can obviously also be considered to write the equations.

#### Probability density function $p_{Tes_N}(.)$: case $N>1$ odd

If $N>1$ is odd, by following the method proposed in Ref.^12^ for the solution of the integral, we obtain

17 $$\begin{aligned}{}&p_{Tes_N}(s;\mu _{t_a},\mu _{t_b},\sigma _a,\sigma _b,N) \nonumber \\&\quad =\int _0^{+\infty }\int _0^{+\infty } p_{Tes_N}\left( \ell _1;\mu _{t_a},\mu _{t_a},\sigma _a,\sigma _a,\frac{N+1}{2}\right) p_{Tes_N}\left( \ell _2;\mu _{t_b},\mu _{t_b},\sigma _b,\sigma _b,\frac{N-1}{2}\right) \delta (\ell _1+\ell _2-s)d\ell _1d\ell _2 \nonumber \\&\quad =p_k(s;\mu _{t_a},\mu _{t_b},\sigma _a,\sigma _b,N)\int _0^1 p_g(w,s;\mu _{t_a},\mu _{t_b},\sigma _a,\sigma _b,N)dw, \end{aligned}$$

where

18 $$\begin{aligned} p_k(s;\mu _{t_a},\mu _{t_b},\sigma _a,\sigma _b,N)= \frac{(\mu _{t_b}+\sigma _b)^{\frac{N-1}{2}}(\mu _{t_a}+\sigma _a)^{\frac{N+1}{2}}}{2^{N-1}\Gamma (\frac{N-1}{2})\Gamma (\frac{N+1}{2})} s^{N-1}e^{-\frac{(\mu _{t_a}+\mu _{t_b}+\sigma _a+\sigma _b)}{2}s}, \end{aligned}$$

and

19 $$\begin{aligned} p_g(w,s;\mu _{t_a},\mu _{t_b},\sigma _a,\sigma _b,N)= \left( 1-w^2\right) ^{\frac{N-3}{2}} \left[ \left( 1-w\right) e^{-\frac{\mu _{t_b}-\mu _{t_a}+\sigma _b-\sigma _a}{2}s w}+ \left( 1+w\right) e^{\frac{\mu _{t_b}-\mu _{t_a}+\sigma _b-\sigma _a}{2}s w}\right] , \end{aligned}$$

and

20 $$\begin{aligned} \int _0^1 p_g(w,s;\mu _{t_a},\mu _{t_b},\sigma _a,\sigma _b,N)dw= \frac{\sqrt{\pi }\,\Gamma (\frac{N-1}{2})}{2^{N-2}\mu ^{\frac{N-1}{2}}} \left[ I_{\frac{N-2}{2}}\left( \frac{s \mu }{2}\right) +I_{\frac{N}{2}}\left( \frac{s \mu }{2}\right) \right] \frac{1}{s^{\frac{N-1}{2}}} \end{aligned}$$

where

21 $$\begin{aligned} \mu =\mu _{t_b}-\mu _{t_a} +\sigma _b-\sigma _a. \end{aligned}$$

Thus,

22 $$\begin{aligned} p_{Tes_N}(s;\mu _{t_a},\mu _{t_b},\sigma _a,\sigma _b,N)&\underset{{\begin{array}{c} N>1 \\ N\, \textrm{odd}\end{array}} }{=} \frac{\sqrt{\pi } (\mu _{t_b}+\sigma _b)^{\frac{N-1}{2}}(\mu _{t_a}+\sigma _a)^{\frac{N+1}{2}} }{2\mu ^{\frac{N-2}{2}} \Gamma \left( \frac{N+1}{2}\right) } s^{\frac{N}{2}} \nonumber \\&\quad \times \left[ I_{\frac{N-2}{2}}\left( \frac{s \mu }{2}\right) +I_{\frac{N}{2}}\left( \frac{s \mu }{2}\right) \right] e^{-\frac{(\mu _{t_a}+\mu _{t_b}+\sigma _a+\sigma _b)}{2}s} \, . \end{aligned}$$

where $I_{n}(x)$ is the modified Bessel function of the first kind.

Note that the method proposed in Ref.^12^ has been developed by imposing some constraints on the parameters $\mu _{t_a}$, $\mu _{t_b}$, $\sigma _a$ and $\sigma _b$. $\sigma _a$ and $\sigma _b$ value. However, it is easy to show that the method remains valid for any value of $\mu _{t_a}$, $\mu _{t_b}$, $\sigma _a$ and $\sigma _b$.

Note also at this point that

23 $$\begin{aligned} p_{Tes_N}(s;\mu _{t_a},\mu _{t_b},\sigma _a,\sigma _b,1)=p_{Tes_1}(s;\mu _{t_a},\sigma _a), \end{aligned}$$

for any $\mu _{t_b}$ and $\sigma _b$ value (in fact they “disappear” for $N=1$), and thus Eq. (22) is also valid for $N=1$.

#### Probability density function $p_{Tes_N}(.)$: case $N>1$ even

If $N>1$ is even, in the same vein, by following the method proposed in Ref.^12^ for the solution of the integral, we get

24 $$\begin{aligned}{}&p_{Tes_N}(s;\mu _{t_a},\mu _{t_b},\sigma _a,\sigma _b,N) \nonumber \\&\quad =\int _0^{+\infty }\int _0^{+\infty } p_{Tes_N}(\ell _1;\mu _{t_a},\mu _{t_a},\sigma _a,\sigma _a,\frac{N}{2}) p_{Tes_N}(\ell _2;\mu _{t_b},\mu _{t_b},\sigma _b,\sigma _b,\frac{N}{2}) \delta (\ell _1+\ell _2-s)d\ell _1d\ell _2 \nonumber \\&\quad =p_k(s;\mu _{t_a},\mu _{t_b},\sigma _a,\sigma _b,N)\int _0^1 p_g(w,s;\mu _{t_a},\mu _{t_b},\sigma _a,\sigma _b,N)dw, \end{aligned}$$

where

25 $$\begin{aligned} p_k(s;\mu _{t_a},\mu _{t_b},\sigma _a,\sigma _b,N)= \frac{(\mu _{t_b}+\sigma _b)^{\frac{N}{2}}(\mu _{t_a}+\sigma _a)^{\frac{N}{2}}}{2^{N-1} \Gamma (\frac{N}{2})^2 } s^{N-1}e^{-\frac{(\mu _{t_a}+\mu _{t_b}+\sigma _a+\sigma _b)}{2}s}, \end{aligned}$$

and

26 $$\begin{aligned} p_g(w,s;\mu _{t_a},\mu _{t_b},\sigma _a,\sigma _b,N)= \left( 1-w^2\right) ^{\frac{N-2}{2}} \left[ e^{-\frac{\mu _{t_b}-\mu _{t_a}+\sigma _b-\sigma _a}{2}s w}+ e^{\frac{\mu _{t_b}-\mu _{t_a}+\sigma _b-\sigma _a}{2}s w}\right] , \end{aligned}$$

and

27 $$\begin{aligned} \int _0^1 p_g(w,s;\mu _{t_a},\mu _{t_b},\sigma _a,\sigma _b,N)dw= \frac{\sqrt{\pi }\,\Gamma (\frac{N}{2})}{2^{N-1} \mu ^{\frac{N-1}{2}} } I_{\frac{N-1}{2}}\left( \frac{s \mu }{2}\right) \frac{1}{s^{\frac{N-1}{2}}} \end{aligned}$$

where

28 $$\begin{aligned} \mu =\mu _{t_b}-\mu _{t_a} +\sigma _b-\sigma _a. \end{aligned}$$

Thus,

29 $$\begin{aligned} p_{Tes_N}(s;\mu _{t_a},\mu _{t_b},\sigma _a,\sigma _b,N) \underset{{\begin{array}{c} N>1 \\ N\, \textrm{even}\end{array}} }{=} \frac{\sqrt{\pi } (\mu _{t_b}+\sigma _b)^{\frac{N}{2}}(\mu _{t_a}+\sigma _a)^{\frac{N}{2}} }{\mu ^{\frac{N-1}{2}} \Gamma \left( \frac{N}{2}\right) } s^{\frac{N-1}{2}} I_{\frac{N-1}{2}}\left( \frac{s \mu }{2}\right) e^{-\frac{(\mu _{t_a}+\mu _{t_b}+\sigma _a+\sigma _b)}{2}s} \,. \end{aligned}$$

Note that, in general, $\int _0^{+\infty } p_{Tes_N}(s;\mu _{t_a},\mu _{t_b},\sigma _a,\sigma _b,N) ds =1, \forall N\ge 1$.

#### Probability density function $p_{s_{\textrm{mix}}}(s;.)$ (single step function)

Let’s first express the pdf $p_{s_N}(s;\mu _{t_a},\mu _{t_b},\sigma _a,\sigma _b,N)$ for a photon to jump over *N* tessels and reach a distance *s*. In practice, the photon stops inside the $(N+1)^{\textrm{th}}$ tessel due to absorption or scattering. See, e.g., an intuitive drawing in Fig. 2 for the scattering case (segment AB where $N=3$). This is obtained as Eqs. (15), (17) and (24)

30 $$\begin{aligned}{}&p_{s_N}(s;\mu _{t_a},\mu _{t_b},\sigma _a,\sigma _b,N) \nonumber \\&\quad =\int _0^{+\infty }\int _0^{+\infty }p_{Tes_N}(s_1;\mu _{t_a},\mu _{t_b},\sigma _a,\sigma _b,N)p_{s_0}(s_2;\mu _t,\sigma _t)\delta (s_1+s_2-s)ds_1ds_2 \nonumber \\&\quad =\int _0^{+\infty }\int _0^{+\infty }p_{Tes_N}(s_1;\mu _{t_a},\mu _{t_b},\sigma _a,\sigma _b,N)p_{Tes_1}(s_2;\mu _t,\sigma _t)\delta (s_1+s_2-s)ds_1ds_2 \nonumber \\&\quad =p_{Tes_N}(s;\mu _{t_a},\mu _{t_b},\sigma _a,\sigma _b,N+1), \end{aligned}$$

where $(\mu _t=\mu _{t_a} \wedge \sigma _t=\sigma _a)$ or $(\mu _t=\mu _{t_b} \wedge \sigma _t=\sigma _b)$, depending if *N* is even or odd, respectively.

Thus, the pdf $p_{s}(s;\mu _{t_a},\mu _{t_b},\sigma _a,\sigma _b)$ for a photon to make a step of length *s*, independently of the number of tessels *N*, is obtained as Eqs. (10) and (30)

31 $$\begin{aligned} p_{s}(s;\mu _{t_a},\mu _{t_b},\sigma _a,\sigma _b)&= \sum _{N=0}^{+\infty }p_{s_N}(s;\mu _{t_a},\mu _{t_b},\sigma _a,\sigma _b,N) {P_{\textrm{N}}}(\mu _{t_a},\mu _{t_b},\sigma _a,\sigma _b,N) \nonumber \\&= \sum _{N=0}^{+\infty }p_{Tes_N}(s;\mu _{t_a},\mu _{t_b},\sigma _a,\sigma _b,N+1) {P_{\textrm{N}}}(\mu _{t_a},\mu _{t_b},\sigma _a,\sigma _b,N). \end{aligned}$$

Equation (31) has been derived for a starting tessel with parameters $\mu _{t_a}$ and $\sigma _a$. However, Eq. (31) also works if we permute $\mu _{t_a}$ with $\mu _{t_b}$, and $\sigma _a$ with $\sigma _b$ in the equation (i.e., we chose the parameters of the starting tessel equal to $\mu _{t_b}$ and $\sigma _b$)

Finally, Eq. (31) allows us to express in general the pdf $p_{s_{\textrm{mix}}}(s;\mu _{t_a},\mu _{t_b},\sigma _a,\sigma _b)$ (single step function) for a photon step of length *s*, for a medium with a probability $1-P$ to have the first tessel with parameters $\mu _{t_a}$ and $\sigma _a$, and probability *P* to have the first tessel with parameters $\mu _{t_b}$ and $\sigma _b$; i.e.,

32 $$\begin{aligned} p_{s_{\textrm{mix}}}(s;\mu _{t_a},\mu _{t_b},\sigma _a,\sigma _b, P )= (1-P) p_{s}(s;\mu _{t_a},\mu _{t_b},\sigma _a,\sigma _b)+P p_{s}(s;\mu _{t_b},\mu _{t_a},\sigma _b,\sigma _a). \end{aligned}$$

In fact, in the previous sections, the theory has been developed for photons that always start from a first tessel of the same type, e.g., “a”. In a binary medium with mixing statistics, the first tessel can have (randomly) different types “a” or “b”. This is why Eq. (32), which represents the average over the two possible types, is necessary. Equation (32) has been implemented in Matlab^®^ language. Note that, to obtain Eq. (32), we did not use the isotropy conditions (Eqs. 4 and 5), thus the pdf $p_{s_{\textrm{mix}}}(.)$ remains valid even if the parameters change with the propagation direction.

### Derivation of the albedo for the random medium with mixing statistics

The albedo for a homogeneous medium is usually expressed as $\mu _s/\mu _t$, where $\mu _s$ is the scattering coefficient, but where, in probabilistic terms, $\mu _s ds$ is interpreted as the probability to be scattered inside a length *ds*. Thus, $\mu _s$ can be seen as the probability of scattering per unit length. In the same way, $\mu _t=\mu _a+\mu _s$ may be interpreted as the probability for a photon to be scattered or absorbed (extinction) per unit length; where $\mu _a$ is the absorption coefficient. In the present case, the “probabilities” $\mu _s$ and $\mu _t$ must be re-calculated, to take into account of the effects of the mixing statistics. To this aim, the probability, $P_{t_{unit}}\Delta s$, to occur in an extinction interaction, inside a very small $\Delta s$, is calculated as:

33 $$\begin{aligned} P_{t_{\textrm{unit}}}\Delta s=\int _0^{\Delta s} p_{s_{\textrm{mix}}}(s';\mu _{t_a},\mu _{t_b},\sigma _a,\sigma _b, P )ds \approx [(1-P)\mu _{t_a}+P\mu _{t_b}]\Delta s, \end{aligned}$$

where we have developed in Taylor series the integral for $\Delta s \ll 1$. Equivalently, the probability, $P_{s_{unit}}\Delta s$, for a photon to occur in a scattering event in $\Delta s$ is expressed as

34 $$\begin{aligned} P_{s_{\textrm{unit}}}\Delta s=\int _0^{\Delta s} p_{s_{\textrm{mix}}}(s';\mu _{s_a},\mu _{s_b},\sigma _a,\sigma _b, P )ds \approx [(1-P)\mu _{s_a}+P\mu _{s_b}]\Delta s, \end{aligned}$$

where we have set $\mu _{a_a}=\mu _{a_b}=0$. Finally, the albedo $\Lambda _{\textrm{mix}}$, to be utilized with the SSF $p_{s_{\textrm{mix}}}(.)$, is obtained as

35 $$\begin{aligned} \Lambda _{\textrm{mix}}=\frac{P_{s_{\textrm{unit}}}}{P_{t_{\textrm{unit}}}}=\frac{(1-P)\mu _{s_a}+P\mu _{s_b}}{(1-P)\mu _{t_a}+P\mu _{t_b}}. \end{aligned}$$

Note that if $\mu _{t_a}=\mu _{t_b}$ and $\mu _{s_a}=\mu _{s_b}$, we obtain the classical albedo for homogeneous media.

### Derivation of the single step function for photons starting from “fixed” positions

For the sake of completeness, in this section we give the essential equations allowing the reader to derive the SSP of photons starting from fixed positions (this topic may be subject of further studies). It has been shown that when $p_{s_{\textrm{mix}}}(.)$ (Eq. 32) is not a pure exponential law, photon steps starting from fixed positions (e.g., from a fixed light source or after the reflection on a medium boudary) must satisfy the following law^1,13^

36 $$\begin{aligned} p_{s_{\textrm{fix}}}(s;\mu _{t_a},\mu _{t_b},\sigma _a,\sigma _b)=\frac{1-\int _0^{s} p_{s_{\textrm{mix}}}(s';\mu _{t_a},\mu _{t_b},\sigma _a,\sigma _b)ds'}{\int _0^{+\infty } s' p_{s_{\textrm{mix}}}(s';\mu _{t_a},\mu _{t_b},\sigma _a,\sigma _b)ds'}, \end{aligned}$$

where $p_{s_{\textrm{fix}}}(.)$ is always a pdf. Using Eqs. (31) and (32), Eq. (36) can be written as

37 $$\begin{aligned}{}&p_{s_{\textrm{fix}}}(s;\mu _{t_a},\mu _{t_b},\sigma _a,\sigma _b)=\frac{1-\int _0^{s} p_{s_{\textrm{mix}}}(s';\mu _{t_a},\mu _{t_b},\sigma _a,\sigma _b)ds'}{\int _0^{+\infty } s' p_{s_{\textrm{mix}}}(s';\mu _{t_a},\mu _{t_b},\sigma _a,\sigma _b)ds'} \nonumber \\ {}&= \frac{1- (1-P) \sum _{N=0}^{+\infty }[F(s;a,b,N+1) {P_{\textrm{N}}}(\mu _{t_a},\mu _{t_b},\sigma _a,\sigma _b,N)]+ P \sum _{N=0}^{+\infty }[F(s;b,a,N+1) {P_{\textrm{N}}}(\mu _{t_b},\mu _{t_a},\sigma _b,\sigma _a,N)]}{(1-P)\frac{\mu _{t_b}+\sigma _a+\sigma _b}{\mu _{t_b}\sigma _a+\mu _{t_a}(\mu _{t_b}+\sigma _b)} + P \frac{\mu _{t_a}+\sigma _b+\sigma _a}{\mu _{t_a}\sigma _b+\mu _{t_b}(\mu _{t_a}+\sigma _a)} }, \end{aligned}$$

where

38 $$\begin{aligned} F(s;a,b,N)=\int _0^{s}p_{Tes_N}(s';\mu _{t_a},\mu _{t_b},\sigma _a,\sigma _b,N)ds'. \end{aligned}$$

Note that the denominator of Eq. (37) is the mean photon step length $\langle s \rangle _{\textrm{mix}}$ in the binary statistical mixture (if $\mu _{t_a}=\mu _{t_b}$ we obtain $\langle s \rangle _{\textrm{mix}}=\frac{1}{\mu _{t_a}}$, as expected). The function *F*(*s*; *a*, *b*, *N*) can be expressed as (see the Supplementary Appendix A for calculation details)

39 $$\begin{aligned}{}&F(s;a,b,N) \underset{{\begin{array}{c} N=1 \end{array}} }{=} 1-e^{-(\mu _{t_a}+\sigma _a)s}, \end{aligned}$$

40 $$\begin{aligned}{}&F(s;a,b,N) \underset{{\begin{array}{c} N>1 \\ N\, \textrm{even}\end{array}} }{=} \nonumber \\&\quad 1 + e^{-(\mu _{t_a}+\sigma _a) s}\sum _{k=1}^{N/2}\frac{s^{k-1}}{\Gamma (k)} \left[ -(\mu _{t_a}+\sigma _a)^{k-1}\right. \nonumber \\&\quad + \left. (-1)^{\frac{N+2}{2}}(-\mu )^{k-N} (\mu _{t_a}+\sigma _a)^{\frac{N}{2}} (\mu _{t_b}+\sigma _b)^{\frac{N-2}{2}}\left( {\begin{array}{c}N-k\\ \frac{N}{2}-k+1\end{array}}\right) \, _2F_1\left( 1,1-\frac{N}{2};\frac{N}{2}-k+2;\frac{\mu _{t_a}+\sigma _a}{\mu _{t_b}+\sigma _b}\right) \right] \nonumber \\&\quad + e^{-(\mu _{t_b}+\sigma _b) s}\sum _{k=1}^{N/2}\frac{s^{k-1}}{\Gamma (k)} \left[ -(\mu _{t_b}+\sigma _b)^{k-1}\right. \nonumber \\&\quad + \left. (-1)^{\frac{N+2}{2}}(\,\mu )^{k-N} (\mu _{t_b}+\sigma _b)^{\frac{N}{2}} (\mu _{t_a}+\sigma _a)^{\frac{N-2}{2}}\left( {\begin{array}{c}N-k\\ \frac{N}{2}-k+1\end{array}}\right) \, _2F_1\left( 1,1-\frac{N}{2};\frac{N}{2}-k+2;\frac{\mu _{t_b}+\sigma _b}{\mu _{t_a}+\sigma _a}\right) \right] , \end{aligned}$$

where $$\left( {\begin{array}{c}.\\ .\end{array}}\right)$$ is the binomial coefficient and $_2F_1(.,.;.;.)$ the hypergeometric function; and

41 $$\begin{aligned}{}&F(s;a,b,N) \underset{{\begin{array}{c} N>1 \\ N\, \textrm{odd}\end{array}} }{=} \nonumber \\ {}&1 + e^{-(\mu _{t_a}+\sigma _a) s} (\mu _{t_b}+\sigma _b)^\frac{N-1}{2} \sum _{k=0}^{\frac{N-1}{2}} \frac{s^{k}}{\Gamma (k+1)} \frac{1}{ (-\mu )^{N-1-k}} \nonumber \\&\quad \times \left[ (-1)^{\frac{N+1}{2}} (\mu _{t_a}+\sigma _a)^{\frac{N-1}{2}} \left( {\begin{array}{c}N-1-k\\ \frac{N-1}{2}-k\end{array}}\right) \, _2F_1\left( 1,k-\frac{N-1}{2};\frac{N+1}{2} ;\frac{\mu _{t_b}+\sigma _b}{\mu _{t_a}+\sigma _a}\right) \right. \nonumber \\&\quad \left. - \frac{(\mu _{t_b}+\sigma _b)^{\frac{N+1}{2}}}{\mu _{t_a}+\sigma _a} \left( {\begin{array}{c}N-1-k\\ -k-1\end{array}}\right)  _2F_1\left( 1,k+1; N+1;\frac{\mu _{t_b}+\sigma _b}{\mu _{t_a}+\sigma _a}\right) \right] \nonumber \\&\quad + e^{-(\mu _{t_b}+\sigma _b) s} (\mu _{t_a}+\sigma _a)^{\frac{N+1}{2}} \sum _{k=0}^{\frac{N-1}{2}} \frac{s^{k}}{\Gamma (k+1)} \frac{1}{ (-\mu )^{N-1-k}} \nonumber \\&\quad \times \left[ (-1)^{k+\frac{N-1}{2}} (\mu _{t_b}+\sigma _b)^{\frac{N-3}{2}} \left( {\begin{array}{c}N-1-k\\ \frac{N-3}{2}-k\end{array}}\right) \, _2F_1\left( 1,k-\frac{ N-3}{2};\frac{N+3}{2};\frac{\mu _{t_a}+\sigma _a}{\mu _{t_b}+\sigma _b}\right) \right. \nonumber \\&\quad \left. + (-1)^{k+1} \frac{(\mu _{t_a}+\sigma _a)^\frac{N-1}{2}}{\mu _{t_b}+\sigma _b} \left( {\begin{array}{c}N-1-k\\ -k-1\end{array}}\right) \, _2F_1\left( 1,k+1;N+1;\frac{\mu _{t_a}+\sigma _a}{\mu _{t_b}+\sigma _b}\right) \right] .\nonumber \\ \end{aligned}$$

In MC simulations, the use of $p_{s_{\textrm{fix}}}(.)$ is fundamental, because it allows one to preserve the invariance property for light propagation and the related reciprocity law.^1^.

### Single step function: direct Monte Carlo simulation

#### Numerical generation of Eq. (32)

Equation (32) has been validated numerically by MC simulation; i.e., by explicitly taking into account each single tessel crossed by the photons. Considering that by definition $p_{s_{\textrm{mix}}}(s;\mu _{t_a},\mu _{t_b},\sigma _a,\sigma _b)$ takes into account only “ballistic” photons (until they are absorbed or scattered), the code has been implemented—in Matlab® language—in the following manner:

1. A uniformly distributed random number $\xi \in \{0,1\}$ is generated;
2. If $\xi <(1-P)$ then the parameters used are $\mu _{t_a}$ and $\sigma _a$, otherwise $\mu _{t_b}$ and $\sigma _b$;
3. A random tessel of length $\ell$ is generated by means of Eq. (2) and the chosen $\sigma _{t_i}$ of point 2 ($i\in \{a,b\}$);
4. A random photon step *s* is generated by means of Eq. (1) and the chosen $\mu _{t_i}$ of point 2 ($i\in \{a,b\}$);
5. If $s>l$ then permute the parameters (i.e., permute a and b parameters) and go to 3, otherwise stop the photon (because absorbed or scattered inside this last tessel) and store the total length traveled and the number of tessels crossed by the photon;
6. Go to 1 until the desired number of photons are propagated.

Note that, usually, in complex MC simulations many photons are launched for a given random tessel configuration. Then, this procedure is repeated a number of times and the “ensemble” average of the results is taken. This approach is imposed by the intensive computation demand of the MC code. However, the same results can be obtained by changing tessel configuration for each launched photon. This is what is done in the present simpler MC context (points 1 to 6 above).

#### Single step function for photons starting at any point inside a tessel

Equation (32) has been derived for photons starting at the medium boundary, and thus also from any tessel boundary (see, e.g., point A in Fig. 2). However, in real MC simulations photons propagate through the medium and steps may start at any point inside a tessel (see, e.g., point B in Fig. 2). In the present context this is not a problem, because Eq. (2) is a memoryless law (exponential pdf), and thus starting from a tessel boundary or inside the tessel does not change the present findings.

**Figure 4:**
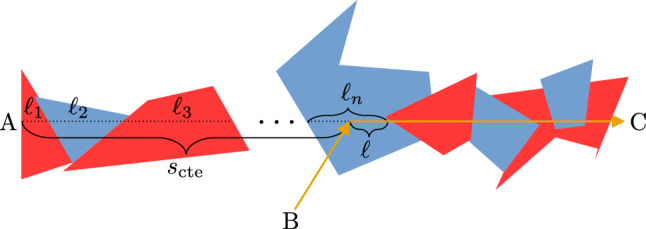
A photon coming from B scatters and goes in direction C. The scattering point is at distance $s_{\textrm{cte}}$ from the boundary, measured along the scattered direction.

To give a more intuitive view on this point, some tutorial MC simulations have been performed. These simulations have the aim to show that Eq. (32) remains valid even in the case where the photons start from points situated inside the tessels. (see, e.g., the $s_2$ step in Fig. 2). Considered that the medium is isotropic, the MC code has been implemented in the following manner (see also Fig. 4):

1. A random $s_{\textrm{cte}} \in [0,+\infty ]$, representing the distance from any point on the medium boundary (e.g., point A in Fig. 4) along the considered direction of photon propagation, is chosen;
2. Random tessel lengths $\ell _1, \ell _2, \ldots$ are generated using Eq. (2), by alternatively using $\sigma =\sigma _a$ and $\sigma =\sigma _b$, until for a given *n*, $\ell _1 + \ell _2+ \cdots \ell _n > s_{\textrm{cte}}$;
3. $$\ell =s_{\textrm{cte}}-(\ell _1 + \ell _2+ \cdots \ell _n)$$ is computed;
4. Points 2 and 3 are repeated many times and the different $\ell$ are saved;
5. The pdf for $\ell$ is obtained by computing the histogram of the $\ell$ data saved in point 4.

In practice, we want to demonstrate that the pdf for $\ell _n$ (see, Fig. 4) is the same as the pdf for $\ell$; i.e., we always have Eq. (2); where $n\in \{1,2,\ldots \}$ represents any $n\textrm{th}$ tessel where the photon is extinct. This means that, it is not important if the photon starts from the tessel boundary or inside the tessel, because Eq. (32) remains the same (i.e., the previous mathematical derivation of Eq. (32) does not change).

## Explanatory examples

In the following subsections we will show the validity of Eq. (32) through some intuitive explanatory examples, and comparisons with MC simulations.

### Example 1

One of the simplest cases we can study is when one of the tessels type (e.g., “b”) has mean length zero (Eq. 3). This means that we remain with an homogeneous medium of type “a” (only sky blue tessels). In this case, we must retrieve the classical exponential law (Eq. 1). Indeed,

42 $$\begin{aligned} \lim _{\sigma _b \rightarrow +\infty } p_{s_{\textrm{mix}}}(s;\mu _{t_a},\mu _{t_b}, \sigma _a,\sigma _b, P )= \mu _{t_a}e^{-\mu _{t_a} s}, \end{aligned}$$

as it is expected. In the case of an isotropic medium, also $\sigma _a$ must go to $+\infty$ because this quantity is linked to $\sigma _b$ by Eqs. (4) and (5), i.e.; $L\rightarrow 0$. In this latter particular case $p_{s_{\textrm{mix}}}(.)$=0, because the mean length of the tessels is zero, and thus the propagating medium does not exist.

### Example 2

Another simple case is when the mean lengths (Eq. 3) of both tessel types are infinite. In this case, we expect that the photon stops inside the first tessel at the entrance of the medium. Considering that tessel types “a” and “b” have probability *P* and $(1-P)$ to be the first tessel, we also expect to obtain the sum of two classical exponential laws (Eq. 1) weighted by their probability. In fact,

43 $$\begin{aligned} \lim _{\begin{array}{c} \sigma _a \rightarrow 0 \\ \sigma _b \rightarrow 0 \end{array}} p_{s_{\textrm{mix}}}(s;\mu _{t_a},\mu _{t_b},\sigma _a,\sigma _b, P )= (1-P)\mu _{t_a}e^{-\mu _{t_a} s}+P\mu _{t_b}e^{-\mu _{t_b} s}, \end{aligned}$$

as it must be. If the medium is isotropic [Eqs. (4) and (5); $L\rightarrow +\infty$] the result remains the same.

Due to the fact that Eq. (43) is not a pure exponential law, photon steps that start from a fixed position must be described using Eq. (36), i.e.;

44 $$\begin{aligned} \lim _{\begin{array}{c} \sigma _a \rightarrow 0 \\ \sigma _b \rightarrow 0 \end{array}}p_{s_{\textrm{fix}}}(s;\mu _{t_a},\mu _{t_b},\sigma _a,\sigma _b)= \frac{\mu _{t_a}\mu _{t_b}}{(1-P)\mu _{t_b}+P\mu _{t_a}}\left[ (1-P)\mu _{t_a}e^{-\mu _{t_a} s}+P\mu _{t_b}e^{-\mu _{t_b} s}\right]. \end{aligned}$$

### Example 3

In the case $\mu _{t_a}=\mu _{t_b}$ and $\sigma _a=\sigma _b$ [see, Eqs. (4) and (5)], we are in the presence of a homogeneous medium, and it is possible to obtain the explicit solution for $p_{s_{\textrm{mix}}}(.)$, as

45 $$\begin{aligned} p_{s_{\textrm{mix}}}(s;\mu _{t_a},\mu _{t_a},\sigma _a,\sigma _a,P)= \mu _{t_a}e^{-\mu _{t_a} s}; \quad 0 \le P \le 1, \end{aligned}$$

where Eq. (45) represents the expected pdf for the homogeneous medium with extinction coefficient $\mu _{t_a}$. The parameter $\sigma _a$ has disappeared because it is no more possible to distinguish the tessels, i.e.; they all have the same optical parameters. It obviously follows, that if the medium is isotropic — i.e.; for $\mu _{t_a}=\mu _{t_b}$ and $\sigma _a=\sigma _b =\frac{1}{2 L}$ [see, Eqs. (4) and (5)], when $P=\frac{1}{2}$—Eq. (45) remains valid.

### Example 4

**Figure 5:**
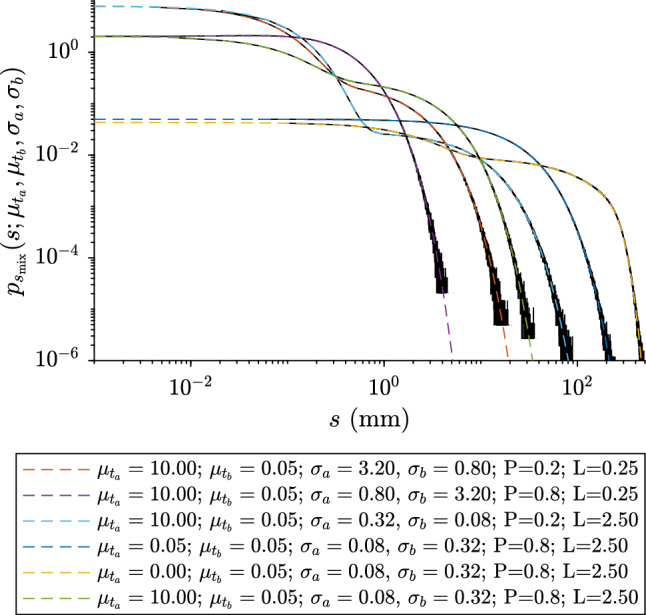
The SSF $p_{s_{\textrm{mix}}}(.)$ as a function of *s* (Eq. 32) for a medium with isotropic Poisson tesselation and binary mixture, and for a set different optical and geometrical parameters. Black lines represent the relative MC data.

To investigate more complex cases, we need to compare our analytical model for the SSF (Eq. 32) with the relative “gold standard” MC simulations. Figure 5 shows the SSF $p_{s_{\textrm{mix}}}(s;.)$ for different optical and geometrical values, compared to the MC simulations. It can be seen that the analytical method gives the same results as the reference MC data.

### Example 5

**Figure 6:**
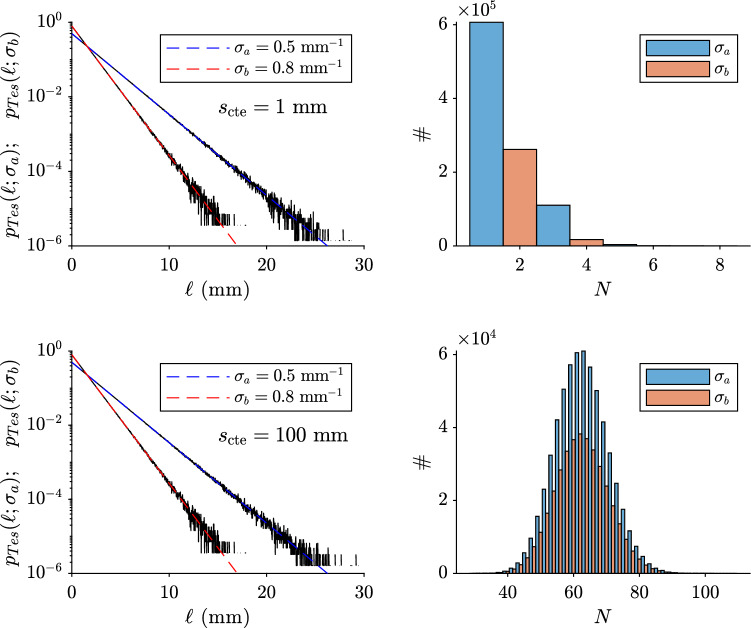
Dashed lines represent Eq. (2). Black lines are MC simulations. The symbol # represents the number of tessels *N* necessary to reach the condition $\ell =s_{\textrm{cte}}-(\ell _1 + \ell _2+ \cdots + \ell _n)$ for any *n* mixed together.

Figure 6 (left panels) shows that the pdf of $\ell$ (see, Fig. 4 for the symbols) is equal to the pdf of $\ell _n$ (Eq. 2). Two representative cases for a small and a large $s_{\textrm{cte}}$ are reported. The data follow a straight lines due to the expected exponential behavior. For a given $s_{\textrm{cte}}$, we also observe two different lines, depending whether we consider tessels of type “a” or type “b”. Hence, MC data for $\ell _n$ have the same pdf as $\ell$ (all possible *n* are mixed on the same line because the behavior is the same). This means that, no matter where a photon starts inside a tessel, Eq. (32) always remains valid. This behavior remains the same for any choice of the parameters (simulations not reported here for obvious reasons).

The panels in the right column of Fig. 6 represent the histogram of the number of tessels *N* necessary to reach the condition $\ell =s_{\textrm{cte}}-(\ell _1 + \ell _2+ \cdots + \ell _n)$ for any *n*. We can see that, as expected, if $s_{\textrm{cte}}$ is small then the probability to have small *N* is also low, and vice versa.

## Conclusions

In the present contribution we have derived the SSF (Eq. 32) for an isotropic Poisson tesselation with a binary mixture model. The SSF allow us to perform MC simulations for any medium geometry, as for the classical Beer–Lambert–Bouguer case, but where the tesselation is built “on the flight”. In other words, once the SSF is known, the binary medium can be treated as a homogeneous medium, and only one MC simulation is in principle necessary to obtain the parameters of interest (instead of hundred of MC repetitions for each random tessel configuration).

Random *s* based on the SSF Eq. (32) can be generated, e.g., as usual, by deriving a look-up table relating $\xi$ and *s* from

46 $$\begin{aligned} \xi =\int _0^s p_{s_{\textrm{mix}}}(s';\mu _{t_a},\mu _{t_b},\sigma _a,\sigma _b) ds'. \end{aligned}$$

The advantage of the look-up table is that it allow us to compute only once, and not at each photon step (actually millions of steps), the infinite sums implicit in Eq. (32).

In principle, by following the flow diagram reported in Fig. 3, the present approach for the SSF derivation can be applied to other stochastic models whose specific “$p_{Tes}(.)$” is known.

Note that, the SSF derived in the present contribution has been obtained for tessels with matched refractive indexes (i.e., all tessels have the same refractive index; not necessarily equal to the external medium), but it would be interesting to know its behavior for the unmatched case. This will be certainly matter for future investigations.

We hope that the present contribution will give to the scientific community a further tool allowing to study photons (particles) propagation in random media.

## Supplementary Information

Supplementary Information.

## Data Availability

The datasets used and/or analysed during the current study available from the corresponding author on reasonable request.
