# Supplementary material for: Probability density function for random photon steps in a binary (isotropic-Poisson) statistical mixture
Source: Sci Rep. 2023 Jun 19;13:9887. doi: 10.1038/s41598-023-36919-2 (PMC10279715; doi:10.1038/s41598-023-36919-2)
Supplement: Supplementary file 1 — Supplementary Information. [file 41598_2023_36919_MOESM1_ESM.pdf]

Appendix A

Calculation details of F(s;a,b,N) [Eq. (38)] in the main text

In this Supplementary Appendix A, using Laplace transform techniques, we provide technical details for the calculation of the integral [Eq. (38)]

$$F(s;a,b,N)=\int_0^s p_{Tes_N}(s';\mu_{t_a},\mu_{t_b},\sigma_a,\sigma_b,N)ds', \tag{A1}$$

where

$$p_{Tes_N}(s;\mu_{t_a},\mu_{t_b},\sigma_a,\sigma_b,N) \underset{N>1}{=} \begin{cases} \frac{\sqrt{\pi}(\mu_{t_b}+\sigma_b)^{\frac{N}{2}}(\mu_{t_a}+\sigma_a)^{\frac{N}{2}}}{\mu^{\frac{N-1}{2}}\Gamma\left(\frac{N}{2}\right)} s^{\frac{N-1}{2}} I_{\frac{N-1}{2}}\left(\frac{s\mu}{2}\right) e^{-\frac{(\mu_{t_a}+\mu_{t_b}+\sigma_a+\sigma_b)}{2}s} & N \text{ even} \\ \frac{\sqrt{\pi}(\mu_{t_b}+\sigma_b)^{\frac{N-1}{2}}(\mu_{t_a}+\sigma_a)^{\frac{N+1}{2}}}{2\mu^{\frac{N-2}{2}}\Gamma\left(\frac{N+1}{2}\right)} s^{\frac{N}{2}} \left[I_{\frac{N-2}{2}}\left(\frac{s\mu}{2}\right)+I_{\frac{N}{2}}\left(\frac{s\mu}{2}\right)\right] e^{-\frac{(\mu_{t_a}+\mu_{t_b}+\sigma_a+\sigma_b)}{2}s} & N \text{ odd}. \end{cases} \tag{A2}$$

We were not able to perform the integral directly, instead we use Laplace transform techniques. We denote by $\mathscr{L}\{f(s)\}=\int_0^{+\infty} dt e^{-st} f(s)$ the Laplace transform of the function $f(s)$ and by $\mathscr{L}^{-1}\{f(t)\}=\int_{c-i\infty}^{c+i\infty}\frac{dt}{2i\pi}e^{st}f(t)$ its inverse Laplace transform. For sake of simplicity, we only treat the case where $N=2p$ is even (the odd case can be handled in the same way). First, we get

$$\begin{aligned}\mathscr{L}\{F(s;a,b,2p)\} &= \frac{\sqrt{\pi}(\mu_{t_b}+\sigma_b)^p(\mu_{t_a}+\sigma_a)^p}{\mu^{\frac{2p-1}{2}}\Gamma(p)}\mathscr{L}\{\int_0^s s'^{\frac{2p-1}{2}} I_{\frac{2p-1}{2}}\left(\frac{s'\mu}{2}\right) e^{-\frac{(\mu_{t_a}+\mu_{t_b}+\sigma_a+\sigma_b)}{2}s'}ds'\} \\ &= \frac{(\mu_a+\sigma_a)^p(\mu_b+\sigma_b)^p}{t(t+\mu_a+\sigma_a)^p(t+\mu_b+\sigma_b)^p},\end{aligned} \tag{A3}$$

thus

$$F(s;a,b,N)=(\mu_a+\sigma_a)^p(\mu_b+\sigma_b)^p\mathscr{L}^{-1}\left\{\frac{1}{t(t+\mu_a+\sigma_a)^p(t+\mu_b+\sigma_b)^p}\right\}. \tag{A4}$$

We seek the inverse Laplace transform by decomposing the fraction $1/(t(t+\mu_a+\sigma_a)^p(t+\mu_b+\sigma_b)^p)$ into simple elements.

$$\begin{aligned}\frac{1}{t(t+\mu_a+\sigma_a)^p(t+\mu_b+\sigma_b)^p} &= \frac{1}{t\,(\mu_a+\sigma_a)^p(\mu_b+\sigma_b)^p} \\ &+\sum_{k=1}^{p}\frac{\sum_{i=0}^{p-k}(-1)^{k+i+1}(\mu_a+\sigma_a)^i(\mu_b+\sigma_b)^{p-k-i}\binom{2p-k}{i}}{(\mu_a+\sigma_a)^{p-k+1}(\mu_a+\sigma_a-\mu_b-\sigma_b)^{2p-k}}\frac{1}{(\mu_a+\sigma_a+t)^k} \\ &+\sum_{k=1}^{p}\frac{\sum_{i=0}^{p-k}(-1)^{k+i+1}(\mu_b+\sigma_b)^i(\mu_a+\sigma_a)^{p-k-i}\binom{2p-k}{i}}{(\mu_b+\sigma_b)^{p-k+1}(\mu_b+\sigma_b-\mu_a-\sigma_a)^{2p-k}}\frac{1}{(\mu_b+\sigma_b+t)^k}.\end{aligned} \tag{A5}$$

One of the sums can be performed by resorting to the hypergeometric function ${}_2F_1(.,.;.;.)$:

$$\begin{aligned}\frac{(\mu_a+\sigma_a)^p(\mu_b+\sigma_b)^p}{t(t+\mu_a+\sigma_a)^p(t+\mu_b+\sigma_b)^p} &= \frac{1}{t}+\sum_{k=1}^{p}\frac{1}{(\mu_a+\sigma_a+t)^k}\Big[-(\mu_a+\sigma_a)^{k-1}+(-1)^{p+1}(\mu_a+\sigma_a-\mu_b-\sigma_b)^{k-2p} \\ &\times(\mu_a+\sigma_a)^p b^{p-1}\binom{2p-k}{p-k+1}{}_2F_1\left(1,1-p;p-k+2;\frac{\mu_a+\sigma_a}{\mu_b+\sigma_b}\right)\Big] \\ &+\sum_{k=1}^{p}\frac{1}{(\mu_b+\sigma_b+t)^k}\Big[-(\mu_b+\sigma_b)^{k-1}+(-1)^{p+1}(\mu_b+\sigma_b-\mu_a-\sigma_a)^{k-2p} \\ &\times(\mu_a+\sigma_a)^{p-1}(\mu_b+\sigma_b)^p\binom{2p-k}{p-k+1}{}_2F_1\left(1,1-p;p-k+2;\frac{\mu_b+\sigma_b}{\mu_a+\sigma_a}\right)\Big].\end{aligned} \tag{A6}$$

Since $\mathscr{L}^{-1}\left\{\frac{1}{t}\right\}=1$ and $\mathscr{L}^{-1}\left\{\frac{1}{(t+x)^k}\right\}=\frac{e^{-xs}s^{k-1}}{\Gamma(k)}$ finally we obtain

$$\begin{aligned}
F(s;a,b,2p) = 1 &+ e^{-(\mu_a+\sigma_a)s}\sum_{k=1}^{p}\frac{s^{k-1}}{\Gamma(k)}\Big[-(\mu_a+\sigma_a)^{k-1}+(-1)^{p+1}(\mu_a+\sigma_a-\mu_b-\sigma_b)^{k-2p} \\
&\times(\mu_a+\sigma_a)^{p}(\mu_b+\sigma_b)^{p-1}\binom{2p-k}{p-k+1}\,{}_2F_1\left(1,1-p;p-k+2;\frac{\mu_a+\sigma_a}{\mu_b+\sigma_b}\right)\Big] \\
&+ e^{-(\mu_b+\sigma_b)s}\sum_{k=1}^{p}\frac{s^{k-1}}{\Gamma(k)}\Big[-(\mu_b+\sigma_b)^{k-1}+(-1)^{p+1}(\mu_b+\sigma_b-\mu_a-\sigma_a)^{k-2p} \\
&\times(\mu_a+\sigma_a)^{p-1}(\mu_b+\sigma_b)^{p}\binom{2p-k}{p-k+1}\,{}_2F_1\left(1,1-p;p-k+2;\frac{\mu_b+\sigma_b}{\mu_a+\sigma_a}\right)\Big],
\end{aligned} \tag{A7}$$

which is the announced result Eq.(40) with $p=N/2$ and $\mu=\mu_{t_b}-\mu_{t_a}+\sigma_b-\sigma_a$.
